# Israeli Acute Paralysis Virus Is an Emerging Pathogen Contributing to Brood Disease of *Apis cerana*

**DOI:** 10.3390/v16091395

**Published:** 2024-08-31

**Authors:** Yanling Xie, Shuai Wang, Yao Liu, Jie Deng, Xiaoling Su, Zhichu Huang, Huoqing Zheng

**Affiliations:** 1 College of Animal Sciences, Zhejiang University, Hangzhou 310058, China; shellyxie@zju.edu.cn (Y.X.);; 2 Jinhua Academy of Agricultural Sciences, Jinhua 321017, China; jhmfyjs@163.com (X.S.);

**Keywords:** *Apis cerana*, diseased brood, pathogens, Israeli acute paralysis virus, artificial inoculation

## Abstract

Larval mortality is the primary symptom of diseased *Apis cerana* colonies, often attributed to sacbrood virus (SBV) and *Melissococcus plutonius.* However, the impact of other common honeybee viruses is frequently overlooked, and their pathogenicity to *A. cerana* remains poorly understood. To investigate the causes of the increasing disease incidence in *A. cerana* brood, we conducted an epidemiological survey, collecting 70 samples from 19 sites across nine provinces in China. Furthermore, we examined the pathogenicity of Israeli acute paralysis virus (IAPV) in *A. cerana* brood through artificial inoculation experiments. Our results demonstrate that, besides SBV and *M. plutonius*, the infection rate and viral load of IAPV in diseased brood are significantly high. Brood artificially inoculated with high concentrations of IAPV exhibited a significant increase in mortality and displayed clinical symptoms similar to those observed in naturally infected colonies. Moreover, a limited resistance to IAPV was observed in *A. cerana* brood, with some individuals able to restrict viral proliferation. Our study highlights the previously unrecognized pathogenicity of IAPV to *A. cerana* brood, demonstrating that IAPV poses a significant threat similar to SBV and *M. plutonius*. We emphasize that IAPV should be recognized as an emerging pathogen causing brood disease in *A. cerana* and managed accordingly in beekeeping practices.

## 1. Introduction

Honeybees are important pollinators of plants in natural and agricultural ecosystems. Multiple factors contribute to the poor health of honeybees, including pathogens, exposure to agrochemicals, and inadequate habitat and nutritional resources [1,2]. Among these, viruses are one of the major threats and cause serious concern for researchers and beekeepers. Some of the most common and harmful viruses in honeybee colonies include acute bee paralysis virus (ABPV), black queen cell virus (BQCV), chronic bee paralysis virus (CBPV), deformed wing virus (DWV), Kashmir bee virus (KBV), Israeli acute paralysis virus (IAPV), sacbrood virus (SBV), and *Varroa destructor* virus-1 (VDV-1) [2]. Over the last decade, our understanding of honeybee viruses has grown dramatically thanks to rapid improvements in and increased accessibility to molecular approaches. However, these advances have mostly been limited to viruses that infect the Western honeybee, *Apis mellifera*.

The Eastern honeybee, *Apis cerana,* is one of the two species of honeybee that have been truly domesticated and used in apiculture. It has adapted to diverse environments and has a broad natural distribution. *A. cerana* is widely found in complex topographic regions with varied habitats, diverse flora, and divergent climates across Asia [3]. Like its western counterpart, *A. mellifera*, *A. cerana* also plays a vital role in agricultural production and biodiversity conservation. However, compared to *A. mellifera*, there is limited research on the health status of *A. cerana*. *A. cerana* was considered to harbor far fewer parasites and pathogens, due to its more strongly expressed collective defense mechanisms [4]. The health of *A. cerana* brood (larvae and pupae) is typically threatened by sacbrood disease caused by SBV, European foulbrood caused by a bacterial pathogen *Melissococcus plutonius*, and the enemy wax moth (*Galleria mellonella*). Although SBV infection does not usually result in colony losses in *A. mellifera*, it poses the greatest threat facing *A. cerana* [5]. SBV can infect both the brood and adult stages of honeybees’ life cycles; however, larvae around two days old are the most sensitive [5].

Besides SBV, many other viruses infect both *A. mellifera* and *A. cerana*, including IAPV. IAPV is a positive-sense, single-stranded RNA virus that can cause systemic infection in honeybees and has been linked with colony losses of *A. mellifera* [2]. In addition to the typical symptom of paralysis and an inability to fly [6], honeybees infected with IAPV exhibit behavioral and cognitive impairments, such as reduced learning and memory abilities [7], reduced lifespan [8], and an exacerbation of the harmful effects of antibiotics [9].

Except for *A. mellifera*, IAPV can also infect other pollinating insects such as *A. cerana* [10,11], bumblebees [12], and wasps [13], as well as various species inside beehives [11,14,15]. IAPV was first detected in Japanese honeybees *A. cerana japonica* in 2011 [10] and in Chinese honeybees *A. cerana cerana* in 2012 [11]. Data from Japan suggest the possibility of inter-specific transmission from *A. mellifera* to *A. cerana* for IAPV infection [10]. A previous study showed that IAPV was the second most common virus found in *V. destructor* in *A. cerana* colonies [16]. Since its first detection, it has been one of the most common viruses detected in *A. cerana*. In a study conducted in 2021 [17], the overall infection rate of IAPV in *A. cerana* reached 18.57%, which was higher than earlier reports [18,19,20], suggesting that *A. cerana* in China may potentially be facing an increasing threat from this virus. 

We collected diseased brood of *A. cerana* from various geographical sources and analyzed the infection levels of *M. plutonius* and seven common viruses. Considering the high prevalence of IAPV in *A. cerana*, we hypothesized that IAPV is another pathogen contributing to larval disease in *A. cerana*. We also inoculated *A. cerana* larvae with IAPV to primarily study the pathogenicity of IAPV on *A. cerana*.

## 2. Materials and Methods

### 2.1. Sample Collection

Seventy samples of diseased *A. cerana* brood were collected between May 2017 and May 2020 from 19 regions in 9 provinces in China (Table 1). Samples were collected only from colonies with dead brood in cells. When sampling, the developmental stages of the majority of brood were identified as larvae. However, in a few cases, the developmental stages of dead brood could not be identified because they had rotted. All the samples were collected in the spring, when brood disease is the most prevalent in *A. cerana* [5]. Each sample consisted of five dead brood, which were placed in a 1.5 mL centrifuge tube and stored in a −80 °C freezer.

### 2.2. DNA Extraction, RNA Extraction and cDNA Synthesis of Diseased Brood

The total genomic DNA of brood samples was extracted using the DNA extraction kit (DP304, Tiangen, Beijing, China), following the procedure suggested by the manufacturer, and resuspended in 50 μL buffer TE. The total RNA of the brood samples was extracted with the RNApure Total RNA Kit (RN-03, Aidlab, Beijing, China), following the manufacturer’s instructions. The quality of each RNA sample was checked using a Nanodrop-2000 spectrophotometer (Thermo Fisher, Waltham, MA, USA). From each RNA sample, 800 ng was taken for cDNA synthesis with the ReverTra Ace qPCR RT Master Mix (FSQ-201, Toyobo, Osaka, Japan), according to the manufacturer’s instructions. The cDNAs and genomic DNA of all samples were stored at −20 °C until use. Genomic DNA was utilized for the qualitative detection of *M. plutonius*, while cDNA was employed for detecting common bee viruses.

### 2.3. Qualitative Detection of Common Bee Viruses and M. plutonius

Normal polymerase chain reaction (PCR) was performed using a high-fidelity enzyme kit (KFX-101, TOYOBO, Osaka, Japan). The total reaction volume of 25 μL consisted of cDNA or genomic DNA (1.0 μL), 2× PCR buffer for KOD FX (12.5 μL), KOD FX (0.5 μL), dNTPs (2 μM, 5 μL), ddH_2_O (4.5 μL), and forward and reverse primers (10 μM, 0.75 μL each) (primer sets listed in Table 2). The cycling profile was 94 °C for 2 min; followed by 40 cycles of 98 °C for 10 s, 56 °C for 30 s, and 68 °C for 1 min; and 72 °C for 5 min. The PCR products were electrophoresed on a 1.5% agarose gel.

### 2.4. Quantitative Real-Time PCR (qRT-PCR) Assays for Viral Load Quantification of Five Bee Viruses

qRT-PCR was performed to investigate the difference in viral loads of five common RNA viruses (BQCV, CBPV, DWV, IAPV, and SBV), given their high prevalence in the diseased *A. cerana* brood. Positive samples of each virus were applied based on the normal PCR detection. The primers for the qRT-PCR are listed in Table 3. The assays were performed in 10 μL volumes containing 1 μL cDNA, 5 μL TB Green Premix Ex Taq II (Takara, Osaka, Japan), 0.5 μL each of the forward and reverse primer, and 3 μL RNase-free water. Amplifications were performed in triplicate with the following PCR conditions: a single cycle at 95 °C for 1 min; 40 cycles at 95 °C for 15 s, 60 °C for 1 min, and 95 °C for 15 s.

Viral loads were quantified using absolute quantification methods according to our former study [17]. Briefly, the linear standard curve equation for each virus was established based on standard curves obtained through six ten-fold dilutions of known amounts of plasmids (pMD^®^18-T Vector, TaKaRa, Osaka, Japan) containing cloned viral target sequences. A linear standard curve was used for each qRT-PCR run. The copy numbers of the viruses were determined by relating the Ct value of each sample to an established standard curve.

### 2.5. Phylogenetic Analysis of IAPV Sequences

Part of the products of IAPV amplification were sequenced (Sangon Biotech, Shanghai, China) for phylogenetic analysis, and the sequences were deposited in GenBank (https://www.ncbi.nlm.nih.gov/genbank/) under the accession numbers MZ494141–MZ494155, accessed on 1 July 2021. These sequences were individually aligned using the ClustalW program in MEGA 6.0 software [30], with other representative homologous sequences retrieved from GenBank. The phylogenetic tree was constructed in MEGA 6.0 software using the neighbor-joining method, based on the Tamura 3-parameter model [31] and a bootstrap value of 1000 replicates. 

### 2.6. Preparation and Quantification of IAPV Solution

Since phylogenetic analysis did not show a species barrier for IAPV infection [17], *A. mellifera* workers were used for the preparation of the IAPV solution. After qRT-PCR detection of the five common viruses as described in Section 2.4, 50 adult *A. mellifera* bees from a colony heavily infected with IAPV were homogenized in 10 mL 1× phosphate-buffered saline (1× PBS buffer). Debris was eliminated using centrifugation (12,000× *g*, 4 °C for 40 min) and virus preparations were prepared using a 0.2-micron filter. Following the method of Remnant et al. [32], 2 μL of the inoculant was injected into the white-eyed pupae via a self-made injector at a slow rate. All the pupae were maintained in an incubator (STIK, Shanghai, China) at 34 ± 0.5 °C, 70 ± 5% relative humidity for 4–5 days. Then, the pupae were used to prepare the viral inoculant using the approach outlined above. qRT-PCR was conducted on the viral inoculant to detect BQCV, CBPV, DWV, SBV, and IAPV. If any viruses other than IAPV were detected, the viral inoculant was injected into white-eyed pupae for further purification. The purification process was repeated several times until only pure IAPV virions were obtained.

### 2.7. Virus Inoculation of A. cerana Larvae

Larvae younger than 24 h (day 1) from healthy *A. cerana* colonies were carefully transferred to 48-well culture plates and fed a total of 150 µL of IAPV-containing food (approximately 1 × 10^6^, 1 × 10^5^, and 1 × 10^4^ genome copy numbers/µL) twice at the following two days, while the control groups were fed the same volume of food with the IAPV solution replaced by PBS buffer. Afterward, the larvae were fed a regular artificial diet (50% royal jelly, 6% glucose, 6% fructose, 1% liquid yeast, and 37% H_2_O) until their pupation (day 6).

### 2.8. IAPV Detection of Inoculated A. cerana Brood

At day 5, 7, 9, 12, 15, and 19, six individuals from both the control group and the experimental group (exposed to a concentration of 1 × 10^6^ copies/μL) were collected and stored at −80 °C for further experiments. Additionally, six dead brood from the 1 × 10^6^ copies/μL experimental group were collected on day 9 and 12. The same inoculation experiment was repeated three times. RNA extraction, reverse transcription, and quantitative detection of IAPV from these samples were performed using the methods described above.

### 2.9. Statistical Analysis

The viral loads were analyzed using one-way ANOVA analysis or/with Tukey’s post hoc test for multiple comparisons. The correlations between the occurrence of viruses were analyzed by Spearman’s rho. The survival curve was analyzed using the Kaplan–Meier test. A *p*-value of <0.05 was considered statistically significant. All statistical analyses were conducted in SPSS 17.0 software.

## 3. Results

### 3.1. Qualitative Detection of Pathogens in A. cerana Diseased Brood

**Figure 1:**
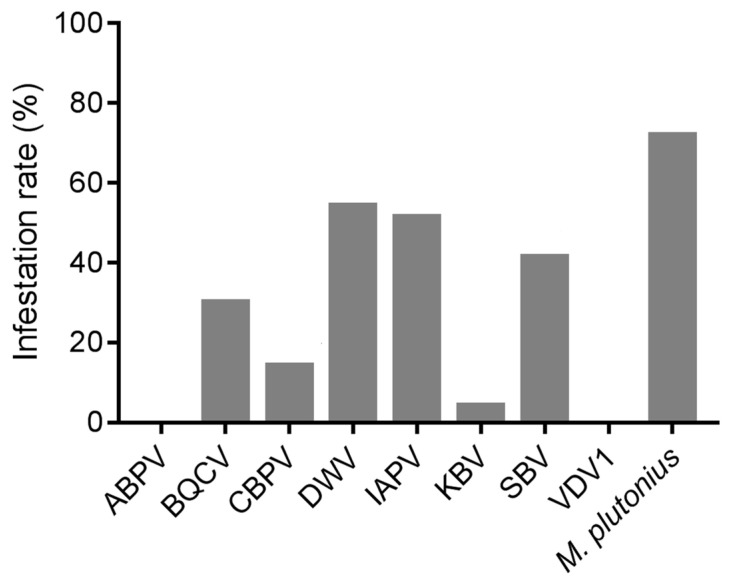
The infection rates of the detected pathogens in the diseased brood of *A. cerana*.

As one of the most prevalent pathogens of *A. cerana* brood, the infection rate of *M. plutonius* was 71.93% among 70 samples of diseased brood (Figure 1). Apart from ABPV and VDV1, neither of which was detected, DWV (54.29%) was the most prevalent virus, followed by IAPV (51.43%), SBV (41.43%), BQCV (30.00%), CBPV (14.29%), and KBV (4.29%).

Among all the samples, 21.43% of the samples were not infected with any bee virus, while almost two-thirds (62.86%; Table 4) were infected with at least two viruses, with a maximum of five viruses simultaneously infecting a sample. Among these viruses, co-infection of BQCV, DWV, and IAPV (8.57%) was the most common, followed by the co-infections of BQCV and DWV; IAPV and SBV; and BQCV, DWV, IAPV, and SBV (each 7.14%). The correlation analysis on their occurrence indicated that IAPV was the virus most strongly correlated with other viruses, showing significant positive correlations with CBPV, DWV, and SBV (Spearman’s rho, *p* < 0.05) (Table S1).

### 3.2. Quantification of Viral Loads in Diseased Brood

**Figure 2:**
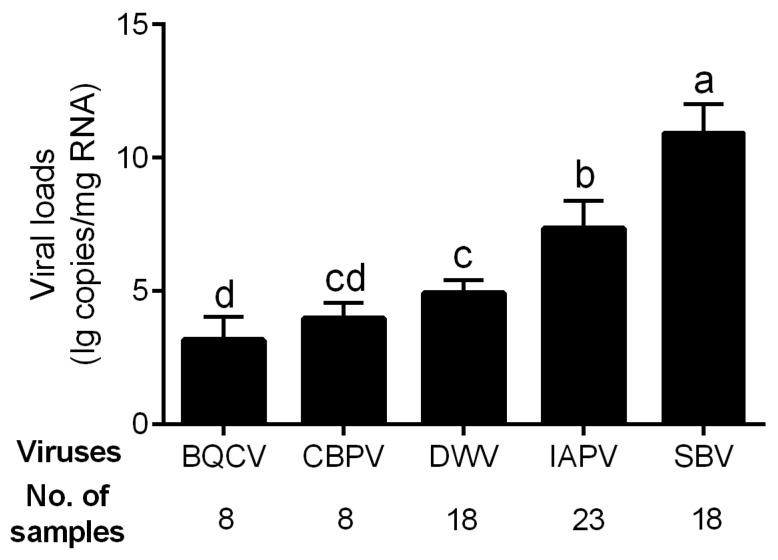
Viral loads of five common viruses in the positive samples of *A. cerana*. Different letters mean significantly different statistics levels (*p* < 0.05).

The viral loads of the five bee viruses with high infection rates, i.e., BQCV, CBPV, DWV, IAPV, and SBV, were determined. The genome copy number of SBV was high in all infected samples, reaching 10.94 ± 1.06 lg copies/mg RNA in average (Figure 2), which was significantly higher than those of other viruses (*p* < 0.01 for all comparisons). The viral load of IAPV in the diseased brood was 7.36 ± 1.02 lg copies/mg RNA on average and significantly higher than those of the other three viruses (*p* < 0.01 for all comparisons). The infection levels of BQCV (3.18 ± 0.86), CBPV (3.98 ± 0.59), and DWV (4.93 ± 0.48) were relatively low.

### 3.3. Phylogenetic Tree of IAPV Isolates

**Figure 3:**
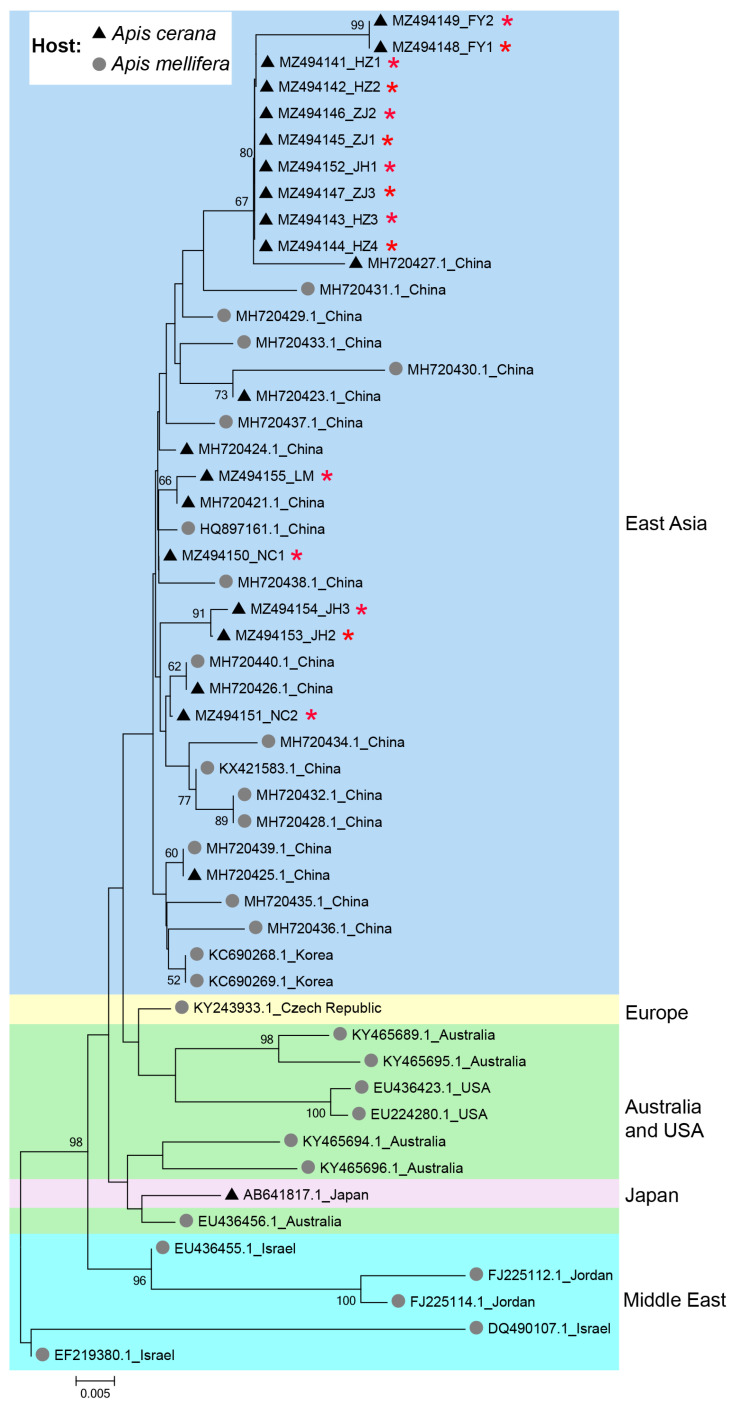
Phylogenetic tree illustrating the genetic relationship of IAPV isolates from different countries and different hosts. The sequences obtained in this study were indicated by red asterisks (*). Numbers at each node represent bootstrap values as percentages of 100; only bootstrap values greater than 50% are shown.

The phylogeny of IAPV clearly showed a geographical pattern, with isolates from nearby countries tending to cluster together. Isolates from *A. cerana* were phylogenetically clustered with those from *A. mellifera*, without forming distinct clades based on host species in China (Figure 3).

### 3.4. Mortality and Viral Load of A. cerana Brood Inoculated with IAPV 

**Figure 4:**
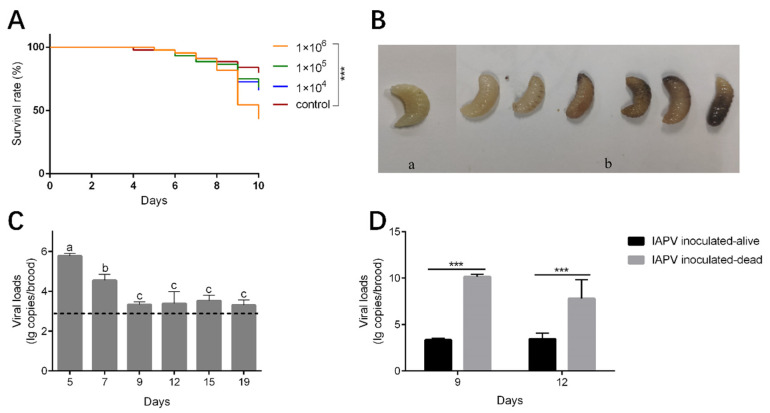
Impact of IAPV inoculation on survival and viral load of *A. cerana* larvae. (**A**) The survival curve of *A. cerana* brood inoculated with IAPV. ***, *p* < 0.001. (**B**) The brood after IAPV inoculation. (**a**) Alive *A. cerana* brood. (**b**) Dead *A. cerana* brood. (**C**) The viral loads of alive *A. cerana* brood after inoculation. The dashed line indicates the mean viral loads of the control groups at the six time points. Low titer of IAPV can be detected in control groups due to the ubiquitous virus in honeybee colonies. Different letters mean significantly different statistical levels (*p* < 0.01). (**D**) The viral loads of alive and dead *A. cerana* brood after IAPV inoculation at day 9 and 12. ***, *p* < 0.001.

The mortality rate of the brood inoculated with IAPV at titers of 1 × 10^4^ and 1 × 10^5^ genome copies/uL was relatively higher, but not significantly different from those of the control group (Kaplan–Meier test, *p* = 0.184 and *p* = 0.244, respectively). However, when inoculated with high titers of IAPV (1 × 10^6^ genome copies/uL), high mortality occurred at day 9 at the prepupae stage (Kaplan–Meier test, *p* < 0.001) (Figure 4A). The dead brood appeared dark brown or milky white, some showing fluid accumulation under the skin or developmental malformations (Figure 4B). These symptoms are similar to those observed in the diseased larvae we collected. The viral load of the survival brood decreased significantly from day 5 to day 9 (Tukey’s post hoc test, *p* < 0.05) (Figure 4C). Meanwhile, the viral loads of dead brood collected on day 9 and day 12 were significantly higher than those of survived brood at the corresponding day ages (one-way ANOVA analysis, *p* < 0.001) (Figure 4D).

## 4. Discussion

The primary symptom of diseased *A. cerana* colonies is larval mortality, commonly attributed to SBV and *M. plutonius*. This attribution often overlooks other viruses present in *A. cerana.* Certainly, the mere presence of pathogens does not necessarily indicate they are the causative agents of the disease, but the prevalence and loads of these pathogens may provide significant clues. Moreover, studies on honeybee viruses have predominantly focused on *A. mellifera,* with limited research addressing the effects of viruses on *A. cerana*. Therefore, it is essential to conduct epidemiological investigations and studies on viral pathogenicity to address the rising incidence of brood diseases in *A. cerana*.

Our results indicated that, among all the detected pathogens, *M. plutonius* was the most prevalent pathogen (Figure 1), while SBV exhibited the highest viral load in diseased *A. cerana* brood (Figure 2). This aligns with previous reports that *M. plutonius* and SBV are the major pathogenic microorganisms threatening *A. cerana* [33,34]. Notably, IAPV warrants attention considering its high prevalence both in *A. mellifera* and *A. cerana* [17]. Among all the tested common honeybee viruses, it showed the second highest prevalence (Figure 1) and viral load (Figure 2) in diseased *A. cerana* brood. When multiple viruses concurrently infect honeybees, IAPV may preferentially proliferate under certain conditions [8,35]. Additionally, IAPV can affect all developmental stages of honeybees, with brood being more susceptible than adult bees [36]. Despite the health status of *A. cerana* being generally better than *A. mellifera*, thanks to its high resistance to the ectoparasite *V. destructor* at colony level, the brood of *A. cerana* is more susceptible to both biological and mechanical stimuli [37,38]. Our survey indicated that IAPV is likely a previously unrecognized pathogen capable of causing disease in *A. cerana* brood.

Phylogenetic analysis indicated that the clustering of IAPV is not influenced by host species but by geographical locations, suggesting that cross-species transmission of IAPV occurs between *A. mellifera* and *A. cerana*, which aligns with previous studies [11,17,19]. This further raises the risk of IAPV to *A. cerana*, since both *A. mellifera* and *A. cerana* are widely kept in Asia; thus, cross-species transmission of the virus may occur frequently [27].

Artificial inoculation of the virus was conducted to further assess the pathogenicity of IAPV to *A. cerana* larvae. A dosage effect of IAPV inoculation by oral administration was observed, with a concentration of 1 × 10^6^ copies/μL of IAPV leading to significant mortality in *A. cerana* brood. A previous study has demonstrated that as few as 10^4^ copies/μL of IAPV can induce mortality in *A. mellifera* pupae within 96 h by injection [8]. This inconsistency can be explained by the different methodologies, the different pathogenicity of IAPV isolates, or the different resistance to IAPV of the two honeybee species. The virus loads of IAPV in dead inoculated brood were 10^6^ times higher than those in alive inoculated brood, suggesting the virus proliferated fast before or after the death of some larvae, while, in others, virus proliferation was inhibited by the hosts. The decrease in viral load from day 5 to day 9 of survived larvae suggest that the host’s innate immune system is triggered and activated to restrict virus replication and clear pathogens [39]. A high mortality rate was observed in the groups of honeybees inoculated with a high titer of IAPV at day 9 before pupation, suggesting that *A. cerana* larvae are more susceptible to IAPV infection at this stage.

To the best of our knowledge, this is the first preliminary study revealing the pathogenesis of IAPV to honeybee species other than *A. mellifera*. This work emphasizes that IAPV should be recognized as an emerging pathogen causing brood disease in *A. cerana*. However, our current knowledge is scarce and further studies are needed to fully elucidate the pathogenesis of IAPV to *A. cerana* and the host defense of *A. cerana* against the virus.

## 5. Conclusions

Our study reveals the previously unrecognized pathogenicity of IAPV to *A. cerana* brood, which partially explains the increasing incidence of brood diseases in *A. cerana*. IAPV has posed a significant threat to *A. cerana*, with high infection rates and viral loads similar to those of SBV and *M. plutonius* in diseased brood. We emphasize that, in beekeeping practice, IAPV should be considered to be an emerging pathogen responsible for brood disease in *A. cerana*. Further studies are necessary to gain a full understanding of the interactions between IAPV and *A. cerana* to finally develop technologies to mitigate the impact of IAPV to *A. cerana*.

## Figures and Tables

**Table 1:** Sampling information of the diseased *A. cerana* brood.

Province	Locality	No. of Samples	Collection Year
Beijing	Haidian (HD)	1	2018
Chongqing	Rongchang (RC)	1	2019
Fujian	Fuzhou (FZ)	1	2017
	Nanping (NP)	1	2019
Guangdong	Longmen (LM)	1	2017
Hunan	Hengyang (HY)	2	2018
Jiangxi	Nanchang (NC)	5	2018, 2019
Shaanxi	Qinling Mountain (QL)	1	2017
Yunnan	Kunming (KM)	4	2018
Zhejiang	Fuyang (FY)	3	2017, 2018
	Hangzhou (HZ)	18	2017, 2019, 2020
	Jinhua (JH)	8	2017, 2019, 2020
	Kaihua (KH)	2	2020
	Lishui (LS)	6	2019
	Longquan (LQ)	4	2018
	Longyou (LY)	1	2019
	Taishun (TS)	2	2018
	Yongkang (YK)	2	2019
	Zhuji (ZJ)	7	2020

**Table 2:** Primer sets of the common pathogens used in this study.

Primer	Pathogen Name	Sequence (5′-3′)	Reference
ABPV-F	Acute bee paralysis virus	TTATGTGTCCAGAGACTGTATCCA	[21]
ABPV-R		GCTCCTATTGCTCGGTTTTTCGGT	
BQCV-F	Black queen cell virus	TGGTCAGCTCCCACTACCTTAAAC	[21]
BQCV-R		GCAACAAGAAGAAACGTAAACCAC	
CBPV-F	Chronic bee paralysis virus	AGTTGTCATGGTTAACAGGATACGAG	[22]
CBPV-R		TCTAATCTTAGCACGAAAGCCGAG	
DWV-F	Deformed wing virus	GTCGTGCAGCTCGATAGGAT	[23]
DWV-R		TTTGCAAGATGCTGTATGTGG	
IAPV-F	Israeli acute paralysis virus	GCGGAGAATATAAGGCTCAG	[24]
IAPV-R		CTTGCAAGATAAGAAAGGGGG	
KBV-F	Kashmir bee virus	GATGAACGTCGACCTATTGA	[25]
KBV-R		TGTGGGTTGGCTATGAGTCA	
SBV-F	Sacbrood virus	GACCCGTTTTCTTGTGAGTTTTAG	[26]
SBV-R		GTGTAGCGTCCCCCTGAATAGAT	
VDV-1-F	*Varroa destructor* virus-1	GCCCTGTTCAAGAACATG	[27]
VDV-1-R		CTTTTCTAATTCAACTTCACC	
Mp-F	*Melissococcus plutonius*	GAAGAGGAGTTAAAAGGCGC	[28]
Mp-R		TTATCTCTAAGGCGTTCAAAGG	

**Table 3:** Primer sets of the quantitative detection of five viruses.

Primers	Pathogen Name	Sequence (5′-3′)	Reference
qBQCV-F	Black queen cell virus	GGAGTCGCAGAGTTCCAAAT	[17]
qBQCV-R		GTGGGAGGTGAAGTGGCTAT	
qCBPV-F	Chronic bee paralysis virus	GGCACCTCAAGATCGTCCAAGTTAC	[17]
qCBPV-R		ACGGAGATGGTGACCTGGTATGG	
qDWV-F	Deformed wing virus	CGTGGTGTAGTAAGCGTCGT	[17]
qDWV-R		TCATCCGTAGAAAGCCGAGT	
qIAPV-F	Israeli acute paralysis virus	TCGCTGAAGGCATGTATTTC	[17]
qIAPV-R		ATTACCACTGCTCCGACACA	
qSBV-F	Sacbrood virus	AACGTCCACTACACCGAAATGTC	[29]
qSBV-R		ACACTGCGCGTCTAACATTCC	

**Table 4:** The multiple infections of viruses in the diseased brood of *A. cerana*.

No. of Viruses	Virus Species	No. of Samples	Ratio/%
0		15	21.43
1	DWV	3	4.29
	SBV	3	4.29
	BQCV	2	2.86
	IAPV	2	2.86
	CBPV	1	1.43
2	BQCV, DWV	5	7.14
	IAPV, SBV	5	7.14
	DWV, SBV	4	5.71
	DWV, IAPV	2	2.86
	BQCV, IAPV	1	1.43
3	BQCV, DWV, IAPV	6	8.57
	DWV, IAPV, SBV	4	5.71
	CBPV, DWV, IAPV	3	4.29
	CBPV, IAPV, SBV	1	1.43
	DWV, IAPV, KBV	1	1.43
	BQCV, DWV, SBV	1	1.43
	IAPV, KBV, SBV	1	1.43
4	BQCV, DWV, IAPV, SBV	5	7.14
	CBPV, DWV, IAPV, SBV	3	4.29
	CBPV, DWV, IAPV, KBV	1	1.43
5	BQCV, CBPV, DWV, IAPV, SBV	1	1.43
Total		70	100.00

## Data Availability

The datasets generated and analyzed during the current study are available upon reasonable request.

## Supplementary Materials

The following supporting information can be downloaded at: https://www.mdpi.com/article/10.3390/v16091395/s1, Table S1: Spearman correlation coefficients among viral detections.
