# Supplementary material for: Israeli Acute Paralysis Virus Is an Emerging Pathogen Contributing to Brood Disease of Apis cerana
Source: Viruses. 2024 Aug 31;16(9):1395. doi: 10.3390/v16091395 (PMC11437426; doi:10.3390/v16091395)
Supplement: Supplementary file 1 [file viruses-16-01395-s001.zip › viruses-3167983-supplementary.pdf]

Table S1. Spearman correlation coefficients among viral detections

| | **BQCV** | **CBPV** | **DWV** | **IAPV** | **SBV** | **KBV** |
|---|---|---|---|---|---|---|
| **BQCV** | 1 | -0.178 | 0.395** | 0.137 | -0.089 | -0.139 |
| **CBPV** | -0.178 | 1 | 0.2 | 0.315* | 0.083 | 0.115 |
| **DWV** | 0.395** | 0.2 | 1 | 0.342* | 0.141 | 0.047 |
| **IAPV** | 0.137 | 0.315* | 0.342* | 1 | 0.327* | 0.206 |
| **SBV** | -0.089 | 0.083 | 0.141 | 0.327* | 1 | -0.029 |
| **KBV** | -0.139 | 0.115 | 0.047 | 0.206 | -0.029 | 1 |

Spearman correlation coefficients (rho) are shown above. $p < 0.05$ (*), $p < 0.01$ (**).
